# The clinical and cost effectiveness of cognitive behavioural therapy plus treatment as usual for the treatment of depression in advanced cancer (CanTalk): study protocol for a randomised controlled trial

**DOI:** 10.1186/s13063-016-1223-6

**Published:** 2016-02-29

**Authors:** Marc Serfaty, Michael King, Irwin Nazareth, Adrian Tookman, John Wood, Anna Gola, Trefor Aspden, Kathryn Mannix, Sarah Davis, Stirling Moorey, Louise Jones

**Affiliations:** Division of Psychiatry, UCL, 6th Floor Maple House, 149 Tottenham Court Road, London, W1T 7NF UK; Research Department of Primary Care & Population Health, UCL Royal Free Site, Rowland Hill Street, London, NW3 2PF UK; Marie Curie Hospice, 11 Lyndhurst Gardens, London, NW3 5NS UK; Palliative Medicine, Royal Free Hampstead NHS Trust, London, UK; Marie Curie Palliative Care Research Department, UCL, 6th Floor Maple House, 149 Tottenham Court Road, London, W1T 7NF UK; Palliative Medicine, Newcastle upon Tyne Hospital NHS Foundation Trust, Freeman Road, Newcastle upon Tyne, NE7 7DN UK; Psychotherapy and CBT, South London and Maudsley NHS Foundation Trust, Institute of Psychiatry, Psychology and Neuroscience, Kings College London, London, UK

## Abstract

**Background:**

The prevalence of depressive disorder in adults with advanced cancer is around 20 %. Although cognitive behavioural therapy (CBT) is recommended for depression and may be beneficial in depressed people with cancer, its use for depression in those with advanced disease for whom cure is not likely has not been explored.

**Methods:**

People aged 18 years and above with advanced cancer attending General Practitioner (GP), oncology or hospice outpatients from centres across England will be screened to establish a DSM-IV diagnosis of depression. Self-referral is also accepted. Eligible consenters will be randomised to a single blind, multicentre, randomised controlled trial of the addition to treatment as usual (TAU) of up to 12 one-hour weekly sessions of manualised CBT versus TAU alone. Sessions are delivered in primary care through Increasing Access to Psychological Care (IAPT) service, and the manual includes a focus on issues for people approaching the end of life. The main outcome is the Beck Depression Inventory-II (BDI-II). Subsidiary measures include the Patient Health Questionnaire, quality of life measure EQ-5D, Satisfaction with care, Eastern Cooperative Oncology Group-Performance Status and a modified Client Service Receipt Inventory. At 90 % power, we require 240 participants to enter the trial. Data will be analysed using multi-level (hierarchical) models for data collected at baseline, 6, 12, 18 and 24 weeks. Cost effectiveness analysis will incorporate costs related to the intervention to compare overall healthcare costs and QALYs between the treatment arms. We will conduct qualitative interviews after final follow-up on patient and therapist perspectives of the therapy.

**Discussion:**

This trial will provide data on the clinical and cost effectiveness of CBT for people with advanced cancer and depression. We shall gain an understanding of the feasibility of delivering care to this group through IAPT. Our findings will provide evidence for policy-makers, commissioners and clinicians in cancer and palliative care, and in the community.

**Trial registration:**

Controlled Trials ISRCTN07622709, registered 15 July 2011.

## Background

Depression is one of the most prevalent mental disorders in people with cancer [1]. It undermines the quality of life for individuals and those close to them [2], may reduce adherence to medication, and may prolong episodes of hospitalisation and increase healthcare costs [3]. Untreated depression is an independent predictor of early death in those with advanced cancer [4].

The prevalence of depression in people with cancer is around 10 % [5] and in advanced cancer may be as high as 21 % [6]. It is possible to treat depression effectively in the general population using antidepressant medication and psychological treatments such as cognitive behavioural therapy (CBT) and interpersonal therapies [7, 8]. However, it is not known whether these approaches are beneficial in those people with cancer who experience depression or those whose low mood exceeds what might be expected in the face of life-limiting illness. A recent systematic review of randomized controlled trials of the efficacy of any treatment intended to reduce depression in adults with diagnoses of both cancer and depression found that only seven relatively small trials have been published [9].

A meta-analysis in 2008 [10] suggested that CBT may be a promising approach [11], although a recent systematic review of interventions for depression in cancer, patients of which are a poor prognostic group, did not generate any well-designed trials where a CBT intervention was used [9]. Indeed, all currently published trials in this field having methodological limitations [10, 12, 13]. The use of specially trained nurses to use CBT skills in palliative care has not been shown to alleviate depressive symptoms in cancer [14]. Whilst some benefit may exist for nurse-led problem solving therapy and behavioural activation for depression in advanced cancer [9], the effectiveness of CBT remains to be evaluated. Evidence exists from qualitative studies with people receiving hospice care that CBT is acceptable [15], but no studies have explored the therapists’ experience of delivering CBT in this context.

The overall economic cost of depression in England was estimated at £9 billion in 2000 [16]. The costs associated with providing standard therapy through IAPT for depression and anxiety is estimated at £750 per patient [17]. Estimated savings are around £1,200 in the first 2 years for the additional GDP produced per person as a consequence of treatment and £300 in NHS costs. The costs attributed to a reduction in suffering, which are measured by the change in quality-adjusted life years, is estimated at £3,300 [17]. Choosing a value of a unit reduction on the BDI-II of greater than £115 as acceptable, CBT is likely to be cost-effective for treating depression in older people in the UK [18]. In a recent UK RCT, the economic evaluation of CBT as an adjunct to care as usual showed that the intervention was cost-effective over 12 months, based on the threshold of £20,000 per QALY used by the National Institute for Health and Care Excellence [19].

A review of the cost-effectiveness of CBT could not draw firm conclusions from the limited data available [20]. Although economic evidence did provide tentative support for the hypothesis that CBT was more efficient than usual care and suggested a modest cost-effective advantage in favour of CBT, none of the reviewed RCTs were in advanced cancer cohorts.

In this paper, we describe the protocol for a national, single-blind, two-arm, randomised controlled trial of up to 12 sessions of CBT offered in addition to usual care through the UK Improving Access to Psychological Therapies (IAPT) service available through primary care, versus usual care alone, for people with advanced cancer who have a DSM-IV diagnosis of depression. We include a nested qualitative component to explore, after final follow-up, the experience of therapy from the perspectives of both participants and therapists. Our data will increase our understanding of the feasibility of delivering this service through IAPT in primary care. Our trial was funded in response to a call from the UK National Institute for Clinical and Care Excellence (NICE) to assess the clinical and cost effectiveness of CBT for depression in this context. Our results will also inform policy-makers advising the London Cancer Alliance and IAPT itself on the suitability of using IAPT for treating those facing cancer long-term or in the advanced stages.

A favourable opinion for the conduct of the study was granted by the London-Camberwell St. Giles NRES committee, Central London REC3 ref 11/LO/0376. This study forms part of the National Cancer Research Network (NCRN) clinical trials portfolio registration number 10255, ISTCRN number 07622709.

### Hypothesis and Objectives

#### Hypothesis

Cognitive Behaviour Therapy plus treatment as usual is more clinically and cost effective than treatment as usual for major depression in adults with cancer that is no longer amenable to curative treatment.

#### Objectives

The objectives of this study are as follows:

1. To determine through a randomised controlled trial the clinical effectiveness of Cognitive Behaviour Therapy plus treatment as usual compared to treatment as usual for depression in adults with cancer which is no longer amenable to curative treatment.
2. To determine through a randomised controlled trial the cost effectiveness of Cognitive Behavioural Therapy plus treatment as usual compared to treatment as usual for depression in adults with cancer which is no longer amenable to curative treatment.

## Methods/design

### Study design

This is a parallel group, single blind, randomised controlled trial comparing treatment as usual (TAU) against TAU plus up to 12 sessions of manualised cognitive behavioural therapy (CBT).

### Population

The study population includes people aged ≥ 18 years with cancer that is no longer amenable to cure, who also have a DSM-IV diagnosis of major depression.

### Location

Recruitment will take place in several sites across England from a variety of clinical services situated in oncology outpatient clinics, primary care clinics or hospices.

Oncology outpatient services where recruitment will take place include those available in 18 hospitals covered by cancer networks in North Central, North East and South East London as well as in the Midlands, West, North East, North West and the South of England. Primary care recruitment will take place through General Practitioner (GP) cancer/palliative care registers through the National Institute of Health Research (NIHR) Primary Care Research Network (PCRN). Participating practices are drawn from the PCRN in areas in which co-applicants are based and where IAPT services are well established and available across the UK. Finally we will also recruit through Marie Curie Hospice Hampstead’s outpatients and day care services.

### Entry criteria for participation in the trial

Inclusion criteria are as follows:

1. People with a diagnosis of cancer not amenable to curative treatment as assessed by their clinician and defined as those receiving palliative radiotherapy, chemotherapy, those with metastatic disease, or subsequent incurable recurrence. This diagnosis will be verified by oncologists or GPs.
2. A DSM-IV diagnosis of depressive disorder using the Mini International Neuropsychiatric Interview (MINI) [21].
3. Sufficient understanding of English, judged by clinic staff, to be able to engage in CBT.
4. Eligible for treatment in an IAPT centre. Either the patient or their GP must be located in an appropriate IAPT catchment area.

Exclusion criteria are:

1. Clinician estimated survival of less than 4 months, verified by the patients’ oncologists or GPs.
2. People with high suicide risk, established through module C of the MINI. The care pathway is described below under risk management.
3. Currently receiving or having received, in the last 2 months, a psychological intervention recommended by NICE aimed at treating depression (for example, Interpersonal Psychotherapy, CBT).
4. Suspected alcohol dependence using the Alcohol Use Disorders Identification Test (AUDIT; [22]).
5. To avoid contamination of the TAU arm by non-study CBT, we will not recruit in areas where the local palliative care service includes routine access to CBT.

### Risk management

When a patient is identified to be at high suicide risk during screening, a clear protocol is in place in which the patient’s key worker is notified and further assessment sought through the regular team. The researcher remains with the patient until additional help is available.

### Participant randomisation

Participants will be randomised to one of two conditions: treatment as usual (TAU) or treatment as usual plus CBT. Randomisation will occur after patients have been assessed to meet the eligibility criteria, have consented to participate and baseline measures have been collected. The trial administrator will send participant details to PRIMENT Clinical Trials Unit (a UKCRC registered CTU) where randomisation will occur using Sealed Envelope, a web-based system. Antidepressants are a predictor of outcome [23]. In cancer, tricyclic antidepressants may be preferentially prescribed over SSRIs because they have fewer relevant side effects, such as nausea, and may be used for both mood and, in lower doses, for pain. Indeed even low doses of tricyclics may be effective [24]. We will therefore stratify our randomisation according to whether or not participants are in receipt of an antidepressant, irrespective of dose.

### Masking

Remaining blind to the treatment group will not be possible for patients or therapists. The researchers and PCRN assessors will be blinded and will be asked to guess group allocation (TAU alone, TAU plus CBT, do not know) at 12 weeks (post-intervention) and 24 weeks (follow-up). Unmasking for those conducting the analysis will not occur until the databases are closed.

### Recruitment methods and procedures

#### From outpatient oncology clinics

NCRN support staff working with UCL researchers will facilitate recruitment from oncology outpatient clinics. Patient GP addresses will be checked to determine that they are eligible to be referred to an IAPT service before they are approached. Either support staff or UCL researchers will then screen suitable patients for depression using the PHQ-2 [25, 26] which includes the first two questions of the Patient Health Questionnaire (PHQ-9) [27], a valid screening measure for depression routinely used in general practice.

Patients who score 3 or more, will be provided with a pre-screening information pack and asked if they would be willing to be assessed for the study. If they score 3 or more but do not wish to participate, their permission will be sought for their GP or oncology team to be informed that they may be depressed. If they agree in principle to take part, a researcher will undertake a further assessment, using the Mini International Neuropsychiatric Interview (MINI) to establish a DSM IV diagnosis of depressive disorder. Screening will be conducted in a place and manner to maximise the privacy of the patient, in an assessment room where this is possible, within the constraints of the clinical environment of each screening centre.

If a DSM IV diagnosis of depression is confirmed, the patient will be given an information pack. The patient will then be given at least 48 hours to decide if they wish to participate in the study before giving written consent for participation. For those who consent to take part, the researcher will then conduct baseline assessments and pass the participant’s details to an independent trial administrator, who will arrange randomisation through PRIMENT Clinical Trials Unit. The study administrator will inform the participant by telephone of their group allocation. For those people randomised to the treatment arm, the administrator will liaise with IAPT to set up the therapeutic sessions. Both administrator and PRIMENT are situated separately from the trial research team to maximise masking of trial arm allocation from the researchers.

#### From GP lists, the PCRN staff

The researcher and PCRN staff will liaise with practice managers and ask them to identify people from their databases who may be eligible for screening. By mutual agreement according to availability at any practice, either the PCRN staff or the researcher will then contact people by telephone or face to face in practices to explore whether they are willing to answer the two PHQ screening questions for depression. The PCRN staff and/or researchers will then follow the same recruitment procedure described above.

#### Self-referral

With the permission of clinical leads in each service, posters and leaflets about the study will be placed in approved oncology clinics and GP practices. The leaflet contains the PHQ-2 for people themselves to conduct a quick assessment of their mood, suggesting to people with a score of 3 or more, that they may have depression and that they should either (a) approach the clinical team within the site or (b) contact the study team directly using the reply slip attached to the leaflet. The process of recruitment will then be the same as that outlined above.

### Description of the Interventions

#### TAU

All participants will receive TAU from all clinicians involved in their care. This consists of routine support such as appointments with GPs, clinical nurse specialists, oncologists and palliative care clinicians. Participants’ physical health and medications will be reviewed and treatment modified according to symptoms such as pain. Psychotropic medication will be prescribed as necessary by either the GP or oncologist. In line with NICE guidance, specific psychological support may be available for those who present with psychological needs at any time, and study participants will not be exempt from receiving external psychological support. We will discourage specific psychological interventions aimed at treating symptoms of depression (for example, CBT or Interpersonal Psychotherapy), but ultimately we cannot interfere with usual care. We will record the numbers of people receiving any psychological therapy during the trial, although the numbers are likely to be small [28].

In cancer, tricyclic antidepressants may be preferentially prescribed over SSRIs because they may be helpful in the management of pain and are less associated with commonly experienced nausea and gastrointestinal problems. Determining the therapeutic dose of antidepressant is complex and even low doses of tricyclic antidepressants may be effective [24]. We cannot stipulate that post-randomisation anti-depressant medication cannot be used or that the dose should be fixed. Withholding a recognised treatment for depression would be unethical and would not reflect treatment as usual.

#### Cognitive Behaviour Therapy (in addition to Treatment As Usual)

The CBT will be delivered through ‘improving access to psychological therapies’ (IAPT) and wellbeing centres. IAPT/wellbeing centres train, supervise and supply therapists to treat people in primary care with mental health problems. For the purpose of the study, only step 3 and step 4 (high intensity) therapists will be used. They will be given a day’s training by the CanTalk team (SM, MS and KM), so their existing CBT skills can be adapted to use a specially developed treatment manual for people with advanced cancer. The manual details modifications in the structure of therapy and its content; in particular, it takes into account physical health problems, existential issues and communication with loved ones.

#### Structure of CBT sessions

NICE recommends 16 to 20 sessions of CBT to treat severe depression in secondary care. Experience shows that in primary care, considerably fewer sessions are taken up. People with advanced cancer may have difficulty coping with longer therapy as their health may be deteriorating. Our intervention will consist of up to 12 sessions of individual CBT delivered face to face or on the telephone over 3 months. Twice weekly sessions may be offered for the first 2 weeks, weekly sessions for weeks 3 to 9 and then 2 sessions in weeks 10 to 12. The timing of the sessions will need to be flexible and pragmatic to fit in with the existing commitments of the IAPT service.

In order to facilitate engagement for those who may not be able to attend sessions face to face, telephone CBT may be offered, providing at least three sessions of face-to-face therapy have already been received. Telephone CBT is already used by IAPT therapists. Moorey, Serfaty, and Mannix will teach CBT therapists how to adapt their CBT techniques for telephone counselling using similar methods to Tutty et al. [29].

#### Content of CBT sessions, guided by a written manual

IAPT guidelines recommend that patients with moderate to severe depression and complex needs receive high intensity (step 3) work. This is consistent with the level 4 psychological interventions recommended by NICE [30] for people with cancer. The CBT intervention will use a flexible approach, adapted for use with people with advanced illness, who face a poor prognosis. The manual was developed by Moorey, Mannix and Serfaty, in which therapists adapted their work to patients with advanced cancer. The key shift is to identifying whether thinking and behaviour are ‘helpful’ or ‘unhelpful’ rather than a reality-testing approach, enabling patients to adopt adaptive strategies to cope with adverse and often unpredictable health circumstances.

The intervention will broadly cover the following:

In *session 1*, an assessment of problems, psychoeducation about depressive disorder and an introduction to the cognitive model will be undertaken. A simple cross-sectional formulation of current emotional distress will be established, and the triggers to emotional distress and how to manage them will be identified, with steps towards one of the patient’s goals. A list of enjoyable activities will be instigated, and unhelpful thinking styles will be identified using specific examples from recent events. *Session 2* will aim to help patients develop an understanding of their problems within a cognitive behavioural framework and begin the process of therapy, using cross-sectional formulation. This will include a discussion of past strengths and coping abilities. Behavioural activation techniques will be used within the constraints of the person’s physical illness. *Session 3* will review the formulation, identifying any new insights/changes. Guided discovery, through a deeper discussion of the patient’s thoughts/beliefs around their illness and their resilience, will help them to apply their resilience under current circumstances. A start will be made on identifying ‘helpful’ versus ‘unhelpful’ thinking and behaviours. *Sessions 4 to 5* will help the patient apply new learning to current difficulties, recent success experiences will be reformulated, and helpful changes will be identified. Guided discovery will be used to help the person notice successful experiences and build resilience. The triggers to emotional distress and strategies for responding will be explored. These will include thought testing and in-session experimentation with allowing intrusive thoughts to pass. *Session 6-7* will focus on thought testing and finding ‘helpful’ alternative thoughts. This will be done within sessions, supplemented by homework completed by the patient between sessions, where logs of patient mood and associated thoughts and behaviours are reviewed. Thoughts and behaviours will then be challenged, and more helpful alternatives considered. Examples of recent success experiences will be added to successes lists and exploration of these for their associated ‘helpful’ thoughts.’ *Session 8* will focus on problem solving and worry time. Confirmation will be made that thought testing/‘helpful’ thoughts concept has been understood. Examples of realistic concerns will be identified to generate a ‘problems to address’ list. An example of one problem will be taken to illustrate the problem-solving approach. The concept of ‘worry time’ will be introduced. *Session 9* will consolidate CBT strategies, reviewing and prioritising a problem list. Planning on how to tackle harder problems will be undertaken, identifying unhelpful thoughts and behaviours with consideration of the pros and cons of potential solutions and the commitment to this process. A review of the use of worry management strategies will also be done. *Session 10* will consist of a review of the person’s perceived progress, including successes and difficulties. *Session 11* will consist of relapse prevention. This will include reviewing presenting difficulties, the progress and personal achievement made, personal resilience and successes, and the development of a relapse prevention checklist. *Session 12* will consist of future planning, reviewing a relapse-prevention checklist, making concrete plans for action if emotional distress recurs or unhelpful behaviours/thinking return. In addition, therapists will be taught on materials contained in three sections in the Manual’s appendix, so, if relevant, these may be addressed with participants. These will include, first, covering existential issues in addition to addressing fears about the mode of dying, fears about the effect of death on others, and fears about what happens after death; second, applying CBT in shorter sessions when health is poor, how to deal with fatigue and coping with loss of function; and third, facilitating communication with partner, families and carers. This will include identifying differing assumptions about attitudes to cancer and illness-related behaviour.

In addition telephone CBT will be implemented. Recent evidence suggests that the use of telephone CBT in the treatment of depression is both clinically and cost effective [30, 31]. We will therefore use this approach where necessary with people who may not be able to continue to attend face-to-face CBT sessions. We anticipate that this situation may arise for some people whose physical condition deteriorates as their cancer progresses. This will improve engagement and minimise attrition.

#### Therapist experience and characteristics

All therapists who work on this trial will be high-intensity therapists. They will have completed a postgraduate diploma in CBT and will have at least 2 years clinical experience post-CBT qualification. These high-intensity IAPT therapists will be familiar with using either cognitive behaviour therapy according to Beck’s model [32] and/or the Seattle Behavioural Activation Programme. Our therapy will use a modified Beck’s model.

#### Additional training for therapists to deliver CBT for patients with advanced cancer

As therapists may not be familiar with the complex needs and existential issues of people with advanced cancer, they will be trained to adapt their skills.

Therapists working on the trial will be required to attend a 1-day training programme on how to apply CBT in this context. A number of the components have already been used for training other healthcare professionals to deliver CBT in advanced cancer or palliative care [14].

### Quality control: adherence to treatment and evaluation of therapy

Therapists will record the components of the therapy that they provide to individual participants using a similar checklist piloted in a previous study [28] but adapted for people with cancer (Table 1). We will encourage all therapists to upload recordings of therapy where possible onto a secure data base using encryption software. Local healthcare trust policy and therapists’ experience of IT systems may limit this process. We will aim to sample 1 in 10 audio recordings of therapy sessions and sample recordings depending on the phase of the intervention (early: sessions 1 to 4, mid: sessions 5 to 8, or late: sessions 9 to 12). Selected sessions will be rated by an accredited member of the British Association of Behavioural and Cognitive Psychotherapists using Revised Cognitive Therapy Scale (CTS-R) [33], which is a reliable measure of the delivery of CBT [34].

**Table 1:** Therapy component checklist

Session the component was covered:	1	2	3	4	5	6	7	8	9	10	11	12
General procedures												
Initial assessment												
Describe Beck’s model and concept of cognitive behavioural therapy (CBT)												
Agree on goals of therapy												
Present a shared formulation												
Goal setting												
Review of shared formulation												
Review of success list												
Relapse prevention/future planning												
Behavioural techniques												
Relaxation training												
Breathing space												
Activity schedule												
Pleasure experiences sheet												
Cognitive techniques												
Refocusing techniques												
Mindfulness												
4-Step process for resilience and coping												
Coping map												
List of strengths and resources												
Reattribution												
De-catastrophizing												
Advantages/disadvantages												
Success list												
Thought diary												
Personal rule (pros/cons)												
Managing worry (worry tree handout)												
Blueprint for coping												
Cognitive-behavioural Techniques												
Guided discovery												
Pleasure prediction sheet												
Pleasure experiences sheet												
Negative triad/negative automatic thoughts												
Applying resilience												
Thinking traps handout												
Reality testing												
Searching for alternatives												
ABC form												
Specific cancer topics												
Impact of physical illness												
Beliefs and expectations about illness												
Plans and hopes for care as disease advances												
Relationship between emotions and physical symptoms												
Concerns about current and future ability to cope												
Concerns about loss of control												
Concerns about accepting help												
Concerns about dying (mode/afterwards/life expectancy)												
Impact of disease and mood on behaviour												
Impact of disease/death on loved ones												
Discussion of ‘the meaning’ of the illness												
Acceptance of unfinished business												

### Therapist supervision and workload

Weekly supervision is a prerequisite of practice within IAPT services and audio-recordings of all therapy sessions is routinely made. Although supervision structures are well set up within IAPT services, SM, KM and MS will be available to discuss any difficulties related to interventions in people with cancer. We anticipate that two IAPT therapists will be required from each Primary Care Trust (PCT) to treat approximately 4.5 participants per year each. We have experience in delivering a training programme for palliative care nurses in CBT skills [35], which improves confidence in managing patients [36]. Relevant sections of this have been adapted to provide CBT therapists with confidence in adapting their skills to people with advanced cancer.

### Measures

#### Screening measures

**PHQ – 2: T**he PHQ-2 [25] consists of the first two questions of the Patient Health Questionnaire (PHQ-9; [27]) a valid screening measure of depression has also been used in cancer services.

The **Mini**-**International Neuropsychiatric Interview** (**M.I.N.I.**) is a short structured diagnostic **interview**, which takes 15 minutes to complete. It was developed jointly by psychiatrists and clinicians in the United States and Europe for DSM-IV and ICD-10 psychiatric disorders [21] and has been widely used in cancer patients.

### Outcome measures

We will collect a number of measures at different time points as shown in Table 2 below.

**Table 2:** Outcome measures and timepoints

Measures	T_3_ Baseline	T_4_ (6 weeks) Mid-intervention	T_5_ (12 weeks) Post-intervention	T_6_ (18 weeks) Follow-up	T_7_ (24 weeks) Follow-up
PHQ-9	✓		✓		✓
BDI-II	✓	✓	✓	✓	✓
EQ5-D	✓		✓		✓
Satisfaction with care			✓		
ECOG-PS	✓		✓		✓
CSRI	✓		✓		✓
Antidepressant	✓		✓		✓
Expectation of therapy	✓				
Blindness			✓		✓
Attrition			✓		✓

### Primary outcome

***Beck Depression Inventory-II*** (BDI-II; [37].

This is a 21-item self-report measure with a maximum score of 63 indicating severe depressive symptoms. It contains few items measuring affective-somatic symptoms, with 15 of the 21 items assessing negative cognitions, which are a target of cognitive interventions. The psychometric properties of the BDI-II are similar to the BDI [38], which is the most widely used self-report instrument for depressive symptoms and has also been used in trials of psychotherapy for people with advanced cancer [13, 39, 40]. The BDI-II also has a number of cognitive elements that are particularly useful for measuring change with CBT.

### Secondary outcomes

#### Patient Health Questionnaire

The Patient Health Questionnaire (PHQ9) [27] screens for depression. It is used in primary care settings, including IAPT services. It has been validated as a measure of depression in primary care [41, 42] and can be administered over the telephone [43].

#### EuroQol

The EuroQol (EQ5D) [44, 45] is a generic utility measure of quality of life consisting of five domains and a visual analogue scale. It will be used in the cost-effectiveness analysis.

#### Satisfaction with care

Satisfaction with care is collected using a visual analogue scale (scored 0 to 10 towards higher satisfaction). This method was used in previous psychotherapy research [46].

#### Eastern Cooperative Oncology Group-Performance Status

The Eastern Cooperative Oncology Group-Performance Status (ECOG-PS) [47] is a scale measuring physical functioning on five levels: 0, asymptomatic normal activity; 1, symptomatic but fully ambulatory; 2, symptomatic and in bed less than 50 % of time; 3, symptomatic and in bed more than 50 % of time; 4, 100 % restricted to bed.

### Measure of service utilisation

#### Client Service Receipt Inventory

The Client Service Receipt Inventory (CSRI) is a short modified version of the CSRI [48], which collects data directly from participants on use of hospital services, day centres, residential homes, rehabilitation centres, paramedic/ambulance, community nurses, occupational/physio therapists, and local GP/practice nurses, as well as social care/social housing use.

### Measures to reduce bias

#### At baseline

##### Antidepressant use

Use of antidepressants may predict outcome and is likely to be correlated with health-services use, such as visits to GP, affecting costs per patient in cost-effectiveness evaluation. We shall record the name and dose of any antidepressants prescribed to participants during the course of the study, as well as any changes in prescribing patterns.

##### Other psychological therapies

We will make a record of any psychological intervention reported by patients or recorded in their case notes during the period of the trial.

(iii) *Expectations at baseline* [49]. Prior to randomization, participants will be asked to predict the degree to which they think their mood will improve or not on a seven-point Likert scale ranging from -3 to +3.

##### Treatment preference

Patients’ preferences for treatment will be collected on a four-point Likert scale (0-3) as in Serfaty et al. [28].

### Post-intervention (12 weeks post-baseline)

#### Non-attendance for CBT

Reason for not attending therapy sessions (for example, did not like therapy, recorded death, etcetera).

#### Patient satisfaction

We shall ask participants to rate on a five-point scale (ranging from not at all to very much) whether they found CBT useful.

### At follow-up (24 weeks post-baseline)

#### Assessment of blindness

Each researcher undertaking assessments will be asked to guess the patient’s trial arm (CBT plus TAU, TAU, do not know).

#### Attrition

Reason for missing follow-up data (for example, too ill, died, etcetera).

### Statistical analysis

#### Sample size and power calculations

Published data for trials of CBT suggest that initial reductions in BDI-II with time may not be linear. A separation in depression scores favouring therapy is observed within 6 weeks of starting treatment [50, 51] and continues after the treatment phase has finished [52, 53].

For ethical reasons, participants are entitled to withdraw from the study without giving a reason; however, we will record the reason for withdrawal from the trial if known. We shall record the timing of any attrition. Differential dropout may occur early on because people are not satisfied with their trial arm allocation. Drop out at later phases is more likely to be due to factors such as death, which are less likely to be influenced by group allocation. Details of the assumptions made are described below and summarised on Table 3.

**Table 3:** Assumptions made about numbers of participants at different timepoints

Time	Cognitive behavioural therapy over treatment as usual; difference in BDI-II score	Percent remaining in the study	TAU	CBT
			*n* = 120	*n* = 120
0 weeks (baseline)	0	100	120	120
6 weeks	3	70	84	84
12 weeks	6	65	78	78
18 weeks	6	63	76	76
24 weeks	6	60	72	72

### Power

(i) *Clinical effectiveness*: Our primary outcome is an overall effect of treatment over the 24-week period of follow-up. We have powered the study to enable detection of a difference in BDI-II of six points (standard deviation 12) between the TAU and CBT groups measured at 12 weeks, assuming a treatment effect of three points after 6 weeks, and a sustained six-point difference after 18 and 24 weeks (Table 1). We have been cautious in assuming a sustained rather than an increasing treatment effect after 12 weeks. Follow-up at 12 weeks post-baseline in other trials ranges from 44 % [14] to over 75 % [12, 13]. Although our client group may be in the last year of life, we do not plan to recruit people who are about to die. We will assume a 70 % follow-up rate after 6 weeks, decreasing to 65 % at 12 weeks and 60 % after 24 weeks (Table 3).

The BDI-II manual reports that the correlation between BDI-II values from sessions 1 week apart is 0.93 (Beck and Steer [37]). To estimate the correlation between measurements 6 weeks apart, the simplest assumption possible is that future values will depend only on the most recent past, and not on any history prior to that (in technical terms, this is called an ‘autoregressive process of order 1’). Then, the correlation between measurements will decay at a constant rate of 0.93 per week, and our best estimate of the correlation between BDI-II measures taken 6 weeks apart is 0.93^6^ = 0.65.

Assuming the attrition rates and correlation reported above, the sample size required to detect an overall difference between the groups, at 90 % power and 5 % significance is 109 participants per trial arm (using a multi-level model adjusting for baseline BDI-II). To account for clustering by therapist, the sample size needs to be inflated by a factor of 1.10. (1 + (average cluster size -1) × intra-class correlation coefficient). This is based on an intra-class correlation coefficient of 0.02 [54, 55] and an average of six participants per therapist post-intervention. We therefore intend to recruit 120 participants per trial arm, with the expected numbers available at each follow-up given in Table 1.

An important secondary outcome is to assess how the treatment effect changes over time.

The proposed sample size will provide 90 % power to detect a six-point difference in BDI-II at 12 weeks and 80 % power to detect a six-point difference at 24 weeks if attrition rates are as stated in Table 3.

### Analysis plan

Clinical data: Analysis of data will be undertaken according to CONSORT guidelines within PRIMENT Clinical Trials Unit. A flow chart will present the follow-up rate for each group, with the reason for non-completion of the BDI-II score. Descriptive results (mean and SD) for demographic and pre and post-treatment BDI-II scores at each follow-up period will be presented by treatment group. Analyses will be on an intention-to-treat basis using multi-level (hierarchical) models. The levels of hierarchy in the data are as follows: first level - repeated measures, second level - participants, and third level - therapist. The primary analysis will test for an overall treatment effect on an intention-to-treat basis using BDI-II over the four follow-ups, controlling for baseline BDI-II score and other baseline confounders, including antidepressant dose. A secondary analysis will also look at the effect of treatment separately at each time point, by including a treatment-time interaction in the multi-level model. A complier average causal effect (CACE) analysis will be performed to take into account the possible lack of adherence to CBT. Analyses will be performed using the current version of STATA (version 13) [56].

Antidepressants will be monitored in order to compare their use between groups to check that they are similar. This will be done by recording all antidepressants prescribed during the trial (name, dose and frequency). This will include SSRIs and other antidepressants such as tricyclics, which are often used in small doses for symptom control in end-of-life care. We will convert antidepressant dose for any drug into a mean equivalent dose of imipramine following a method we used in another trial of CBT for depressed older people [28].

### Analysis of economic data

Resource use and costs: Our trial population will have serious physical illness, with multiple hospital contacts for necessary physical treatments recommended by clinicians. It is therefore unlikely that hospital utilisation for physical reasons will differ significantly between the two arms, and this is therefore not being recorded to minimise patient burden. We will, however, record the number of contacts with community services, including mental health, especially as these are most likely to be influenced by our intervention. Therefore, the CSRI will be modified to collect data on community health services only. Resource utilisation will be multiplied by unit costs [57, 58] to estimate individual-level care-package costs for each patient at baseline and over the trial period. Costs related to the intervention will be included. We will calculate mean cost of CBT per participant, the incremental health and social care cost, the incremental QALY gain and incremental cost-effectiveness ratio. Results will be analysed on an intention-to-treat basis. Using multivariate regression methods, we will explore differences in costs and outcomes between treatment groups. We shall adjust for need-related factors, including functionality (assessed using BDI-II).

Bootstrapped incremental cost-effectiveness ratios and acceptability curves will be estimated in order to relate differences in costs and outcomes between the groups. Capturing benefits over a patient’s lifetime is an important issue, given outcome data will be missing at the end of the trial. We will use assumption-based modelling techniques [58] to explore the cost-effectiveness of the intervention using a lifetime horizon. In order to assess the key thresholds at which the intervention would be cost-effective, threshold analysis, for example, may be used as one method of framing assumptions around transition probabilities. The reliability of the model will depend on the quality and extent of missing data from the trial.

### Qualitative data

We will use purposive sampling and write to 20 participants in the trial who received CBT at the end of their follow-up period to invite them to take part in qualitative interviews to explore their experience of the trial and their therapy. We will also interview 12 therapists to explore their experience of delivering therapy to this population. Semi-structured, one-to-one interviews will be conducted using a topic guide either in the participants’ homes or in the department where the research is taking place. All interviews will be audio recorded for transcription at a later date. Data will be analysed using thematic content analysis. Findings from these data will be used to generate recommendations on what may be improved in the delivery of CBT to people with advanced cancer.

### Ethical approval

This trial has been approved by the London-Camberwell St. Giles Research Ethics Committee, part of the NHS Health Research Authority (reference 11/LO/0376). The trial will be conducted in compliance to the Declaration of Helsinki. Informed written consent will be obtained from all participants.

## Discussion

This trial will provide robust evidence on the effectiveness and cost-effectiveness of individual cognitive behaviour therapy for treating people with advanced cancer and depressive disorder. Cancer will affect one in three of the UK population during their lifetime, and it remains one of the commonest causes of death. In caring for people with advanced cancer, much effort may be expended on addressing treatments for physical problems including the management of symptoms such as pain, nausea and fatigue that are common as health deteriorates. Good supportive care at this time should include accurate assessments of mental health, and diagnosis and treatment for depression when necessary. If found to be effective, use of talking therapies may enhance the experience not only of those people facing advanced cancer but also those close to them.

This trial will generate data to aid our understanding of the feasibility of delivering CBT to a population with deteriorating physical health in the community through the existing network of IAPT therapists. We shall gain information on the interfaces between oncology services in secondary care, CBT in primary care and the hospice in the third sector. Some day care services provided through hospices have the capacity to offer talking therapies within current models of care [59, 60]. Evidence from this trial may inform the likely benefits of strengthening these services within palliative care, as well as assess whether augmenting usual care with CBT is cost-effective based on the National Institute for Health and Care Excellence threshold.

Analyses of the content of therapy sessions will enhance our understanding of the issues of importance to people with advanced cancer who experience depression. Our data may reveal domains of worry and psychological need in those facing the approach of death with which we are not yet familiar. Such new knowledge may enable the development of recommendations and guidance for therapists providing CBT in this context, and for the wider clinical community caring for people with cancer for whom cure is not likely.

### Trial status

Participant recruitment began on 19 September 2012 and is ongoing.

## Abbreviations

CBT: Cognitive Behavioural Therapy
IAPT: Improving Access to Psychological Therapies
NIHR: National Institute for Health Research
PCRN: Primary Care Research Network
